# Multivariate approaches to behavioral physiology

**DOI:** 10.18632/oncotarget.16612

**Published:** 2017-03-27

**Authors:** Maurizio Casarrubea, Andrea Santangelo, Giuseppe Crescimanno

**Affiliations:** Department of Biomedicine and Clinical Neurosciences, Laboratory of Behavioral Physiology, Human Physiology Section “Giuseppe Pagano”, School of Medicine, University of Palermo, Italy

**Keywords:** cluster analysis, stochastic analysis, adjusted residuals, T-pattern analysis, behavioral neuroscience

During the last decades, studies in the field of behavioral neurosciences have been, in some extent, quite conservative in the renewal of their methods and approaches. Such an issue has been discussed in various papers. For instance, an elegant review by Kalueff and Colleagues [1] highlighted an unfortunate and persisting association: on the one hand, the lack of converging findings and, on the other hand, the lack of new/alternative approaches to study anxiety and depression. Actually, with thousands of published papers so far, the largest amount of behavioral studies on depression, on anxiety and, more in general, on behavioral neurosciences, utilizes the evaluation of quantitative parameters of individual components of the behavior (e.g., frequencies, durations, percent distributions, latencies, etc). On this subject, paradigmatic might be the instance of a well-known experimental apparatus mainly used to assess anxiety in rats and mice, namely, the hole-board. Basically, a hole-board is an enclosed wooden or plastic arena provided with a variable number of holes in the ground, where the rodent can insert its head. Utilization of hole-boards orbits around the head-dip (the insertion of the head into one of the holes) and around the basic premise that high anxiety levels should reduce head-dip, while low anxiety levels should increase it. Nonetheless, diverging findings surround this key component of the response to anxiety in hole-board. Such a lack of consensus, well underlined by Brown and Nemes [2], emerges also from our researches. For instance, on the one hand we have not observed significant changes of head-dip frequencies following anxiolytic treatment [3], on the other hand, the administration of a potent anxiety-inducing molecule provoked evident increases of head-dip count [4]. It goes without saying that simple quantitative measures of head-dip alone should be avoided or, at least, very prudently used. Consistently, we showed that when head-dip is evaluated in terms of its relationships with another component of the rodent's repertoire, namely the sniffing of the edges of the holes (edge-sniff), the effects of anxiolytics and anxiety-inducing molecules become behaviorally coherent [3, 4]. The explanation of such an outcome probably lies in the emotional/motivational load underlying the relationship between the sniffing of the hole-edges and the insertion of the head inside the holes [3, 4]. In brief, we hypothesized that the transition from edge-sniff to head-dip has not the same weight of the opposite transition, from head-dip to edge-sniff: the former transition, indeed, representing the shift from the exploration of the border to the insertion of the head inside, would be heavily dependent on animal's motivation to explore and, as such, heavily influenced by anxiety level and related pharmacological manipulations [3, 4]; on the contrary, the latter transition (from head-dip to edge-sniff), representing the conclusion of the hole-exploration process would be, presumably, much less influenced by anxiety level. This example, concerning the analysis of the existing patterning between head-dip and edge-sniff calls a stimulating topic of discussion. Indeed, if the assessment of a simple bi-variate patterning is able to highlight aspects otherwise undetectable, what happens when the comprehensive behavior of the subject is evaluated in terms of structure, i.e., relationships among its components?

**Figure 1:**
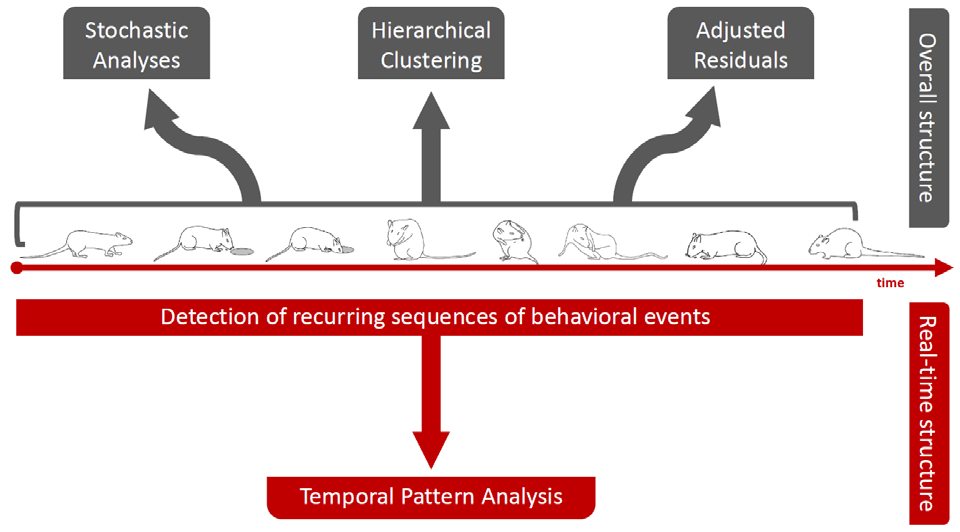
Example of four different multivariate approaches to the study of behavior. Stochastic analysis, cluster analysis and adjusted residuals can be utilized to obtain information about the comprehensive structure of behavior; temporal pattern (T-Pattern) analysis can be used to gain information concerning the real-time structure of behavior.

Usually, the study of behavior requires the utilization of an ethogram, namely, a list of individual components of the subject's repertoire and their description. These discrete components can be easily defined by means of quantitative assessments such as frequencies, percent distributions, durations etc. Nonetheless, the possibility to quantify each behavioral component, alone, through even hundreds of numbers does not automatically imply the opposite possibility to use those numbers to figure out what the behavior is in its functional uniformity. It is our contention that a given behavior can be understood, from a functional perspective, only if the relationships among its constitutive components are assessed. The reasons for this rely on the meaning of the word “function“ and in its teleological implications. Actually, the function of a system–its aim in physiological perspective–emerges from the relationships between the elements of the system itself and, as such, can be understood exclusively taking into account these relationships. Hence, quantitative approaches to the study of behavior should be partnered, whenever possible, with techniques able to detect these relationships. These analytical tools belong to the realm of multivariate analyses. Examples are represented by hierarchical clustering [4], stochastic analysis [5], adjusted residuals [6], T-pattern analysis [7, 8]. Figure 1 presents a synopsis concerning the application of various multivariate approaches to the study of behavior. By using cluster analyses, it is possible to analyze subject's activity in terms of similarities among components, the stochastic approach provides information about the existing probabilistic relationships, adjusted residuals provide the statistical weight of each transition; finally, T-patterns provide information on the real-time structure of the ongoing behavior as they represent sequences of events sharing significant constraints among the interval length separating them. Overall, the usefulness of these approaches lies just in the possibility they offer to consider the relationships between the elements of the behavior, leading the researcher to the assessment of outcomes, greatly beyond what is intuitively deducible by means of observation and/or by means of conventional quantitative evaluations. We believe that the opportunity to study a given behavior both in terms of quantitative features and structural dynamics is able to open new perspectives in the evaluation of the effects induced by the administration of existing and/or new molecules.
